# Implementation of a recovery-oriented assertive community treatment (Re-ACT) program for people with heavy use of psychiatric treatment in Switzerland: results from a three-year pilot study

**DOI:** 10.1186/s12888-025-07287-0

**Published:** 2025-08-27

**Authors:** Mariela E. Jaffé, Julian Moeller, Franziska Rabenschlag, Christine Althaus Aebersold, Jörg Eysell, Constantin Bruttel, Lukas Imfeld, André Nienaber, Undine E. Lang, Christian G. Huber

**Affiliations:** 1 Universitäre Psychiatrische Kliniken (UPK) Basel, University of Basel, Basel, Switzerland; 2 Faculty of Psychology, Center of Social Psychology, University of Basel, Basel, Switzerland; 3 Faculty of Psychology, Division of Clinical Psychology and Epidemiology, University of Basel, Basel, Switzerland; 4 ZI, Mannheim, Germany

**Keywords:** Assertive community treatment, High utilization, Recovery, Relapse prevention, Revolving door, Severe mental illness

## Abstract

**Background:**

International guidelines recommend the provision of community based treatment as an alternative to predominant inpatient stays in psychiatric hospitals for people with severe mental illnesses. Assertive community treatments have been introduced across the globe; however, the development and implementation of such treatment options are still limited in Switzerland in general and were, until recently, not available in Basel-Stadt.

**Methods:**

We here describe the development and implementation of an assertive community treatment program created specifically for people with previous heavy use of psychiatric inpatient services in the canton of Basel-Stadt. The program offers this patient group a need-centered and recovery-oriented treatment option following an inpatient stay. Primary objectives are a reduction in the likelihood of further inpatient stays and the potential experience of involuntary admissions as well as high participant satisfaction.

**Results:**

Results from the three-year pilot study (2019–2022) are reported here on a case-level. We compare objective outcome measures for people participating in the program after an inpatient stay and receiving minimal treatment (*n*_*cases*_ = 110) versus people not participating (*n*_*cases*_ = 292). Overall, we show that program participation is associated with a lower number of inpatient treatment days, inpatient stays and number of involuntary admissions. Feedback on subjective outcome measures indicates high treatment satisfaction.

**Conclusion:**

These findings speak to the feasibility of the program’s implementation and provide a first outlook for a new recovery-oriented treatment option for people with a history of frequent hospitalizations.

## Introduction

What happens after an inpatient stay at a psychiatric hospital? Patients are discharged, ideally after feeling substantially better, and return home. Eventually, outpatient treatment is organized or an effective support system is set up that aids them with the transition of going back to their daily life. However, patients with severe mental illnesses and repeated psychiatric hospital stays might face a different situation. Handling a severe mental illness that is potentially also associated with higher risks of medical conditions, for example cardiovascular diseases [1], and needs related to social connections, family, work, or a lack of general activities can be stressful, overwhelming, and destabilizing – especially when changing from a highly structured environment in a hospital to a home setting [2]. If the mental health condition worsens or further stressors are experienced, patients may then relapse and need to be readmitted to a psychiatric hospital [3, 4]. These frequent alternations between settings and reoccurring stays at a psychiatric hospital can result in further unmet needs and destabilization. To break this cycle, need-adapted, time-flexible treatment is required outside of the hospital, ideally in the homes of patients [5, 6]. In Basel-Stadt, Switzerland, such a program did not exist until recently. In 2019, a pilot project was implemented to deliver a newly developed recovery-oriented assertive community treatment (Re-ACT) to patients with severe mental illnesses and a history of high utilization of psychiatric inpatient treatment. A study was conducted to evaluate the project’s implementation and preliminary treatment outcomes.

### Background

Psychiatric guidelines recommend providing treatment in the least restrictive and stigmatizing environment possible and highlight assertive community treatment (ACT) at home as a first line service to support people with severe mental illnesses (e.g., S3-guideline on psychosocial therapy by the Deutsche Gesellschaft für Psychiatrie und Psychotherapie, Psychosomatik und Nervenheilkunde e.V. (DGPPN; German Association for Psychiatry, Psychotherapy and Psychosomatics) [7], or the National Institute for Health and Care Excellence (NICE) guidelines for psychosis and schizophrenia, [8]). These treatment models are evidence-based [9, 10]: They often entail treatment by a multidisciplinary team, which offers home visits and covers not only medical treatment but also fosters inclusion in the community and provides need-adapted treatment plans to improve the psychosocial well-being of the person [6, 9, 11].

Assertive community treatment has been introduced worldwide (for an overview, see [9, 12], for specific country cases see [13, 14]) and studies have shown positive effects regarding psychiatric hospital use, housing stability, symptom improvement and quality of life ([2, 9], see also [15]). However, assertive community treatment can include a variety of setups with different care intensity levels and different target groups and other research [16] does not show an advantage over other forms of community care (e.g., standard community care).

Until recently, no such program was available for patients in and around the University Psychiatric Clinics Basel, a psychiatric hospital that offers inpatient and outpatient mental health services in a catchment area in the German-speaking part of Switzerland. In 2019, a project group implemented Re-ACT, which included continuous outpatient treatment at home starting directly after an inpatient stay. The objective of this program was to support the transition from hospital to home and to offer a recovery-oriented and need-adapted outreach service. This program was developed in parallel to a shorter, transitional intervention delivered by the same project team (see [17]).

We here present the first results of the 3-year pilot project from the implementation of the recovery-oriented assertive community treatment (Re-ACT) at the University Psychiatric Clinics Basel, Switzerland. These findings describe the feasibility of the program’s implementation by reporting the development of the project team and the numbers of cases handled. They further provide a glimpse of objective outcomes such as changes in numbers of inpatient days in the follow-up period and subjective outcomes such as participants’ satisfaction.

## Method

### Recovery-oriented assertive community treatment (Re-ACT) in Basel-Stadt

The Re-ACT program by the University Psychiatric Clinics Basel was designed to support patients with severe mental illnesses who had a history of high utilization of inpatient treatment. Patients were able to join the program after an inpatient stay and would then receive recovery-oriented support at home for as long as indicated and requested. To do so, the team would assess individual unmet needs using a structured questionnaire (the Camberwell assessment of need short appraisal, [18, 19]) across the different dimensions of recovery (intrapersonal, relational, social, [20]).The health care professional would then discuss unmet needs with the program participant and both would agree on individual treatment objectives and a treatment plan together.

A multidisciplinary team consisting of psychiatrists, social workers and nurses delivered the treatment. Topics that were covered in the treatment included handling of the illness, building social relationships, partnerships, family, sexuality, living situation, life, work and structuring one’s day. The team supported patients with their recreational activities and around questions on finances and administration. To further strengthen the relational and social processes and to support the program participant’s recovery in the community [20], the team would also include their family, friends, or employers in the meetings, if the participant agreed.

Office hours of the program covered all workdays (Monday to Friday) from 8am to 5pm. Off hours and weekends were covered by the standard services of the University Psychiatric Clinics Basel that provide services 24/7/365, including telephone counselling and personal appointments at the hospital’s central admission unit. These services have access to the electronic healthcare documentation of the Re-ACT program and can organize subsequent contacts with the Re-ACT team as soon as they are available again.

The costs for participating in the program were covered by health insurance and the canton of Basel-Stadt. In Switzerland, the health insurance system is based on mandatory individual coverage through regulated private health insurers (private companies). For people with a low income, the government provides subsidies. Individuals can freely choose their insurer and can switch between companies; the companies must cover the same standard benefit package, which is regulated by federal legislation [21, 22].

The pilot project was implemented from April 2019 to March 2022. During this time, numerous patients participated in Re-ACT. However, the COVID pandemic and local lockdowns affected its implementation (see also [23, 24]). Switzerland only had a few lockdowns and the project team was able to organize patient meetings via telephone or videoconference during periods with contact restrictions. However, they were able to resume in-person meetings quickly following respective safety measures. Team internal meetings, including supervision sessions, were shifted to videoconferences.

### Human ethics and consent to participate declaration

Ethics approval from swissethics/Ethikkommission der Nordwest- und Zentralschweiz (EKNZ; ethics committee for northwest and central Switzerland) with the ID 2019 − 00531 was obtained for the program’s evaluation. With the approval of the local ethics committee, anonymized clinical routine and administrative data from inpatients of the University Psychiatric Clinics Basel were used to evaluate the Re-ACT program as well as to generate data for a comparison group of persons who did not agree to participate in Re-ACT. The requirement to collect informed consent was waived, as only already available routine data generated during clinical treatment were used for the analyses. All procedures were carried out in conformance with the Declaration of Helsinki in its latest revision.

### Program inclusion criteria for participants and procedure

The target group of the Re-ACT program was 18 to 63 year old patients living in the canton of Basel-Stadt with a history of utilization of psychiatric services that could be categorized as high [25, 26]. For the Re-ACT program, high utilization was conceptualized as having at least 180 inpatient days and/or at least 3 inpatient stays (no minimum duration, but the team checked the records of the previous treatment) at the psychiatric hospital during the last 2.5 years. Patients with all diagnoses were included and eligible to join the program. However, while patients with a primary diagnosis of addiction or dementia were not primarily targeted, they could be included on a case-by-case basis. Exclusion criteria for the study were being younger than 18 years old, insufficient knowledge of German to communicate with the Re-ACT team, and living in social psychiatric accommodations with community treatment like care structures. Additional exclusion criteria for the Re-ACT program was declining participation (see the design section below).

**Fig. 1:**
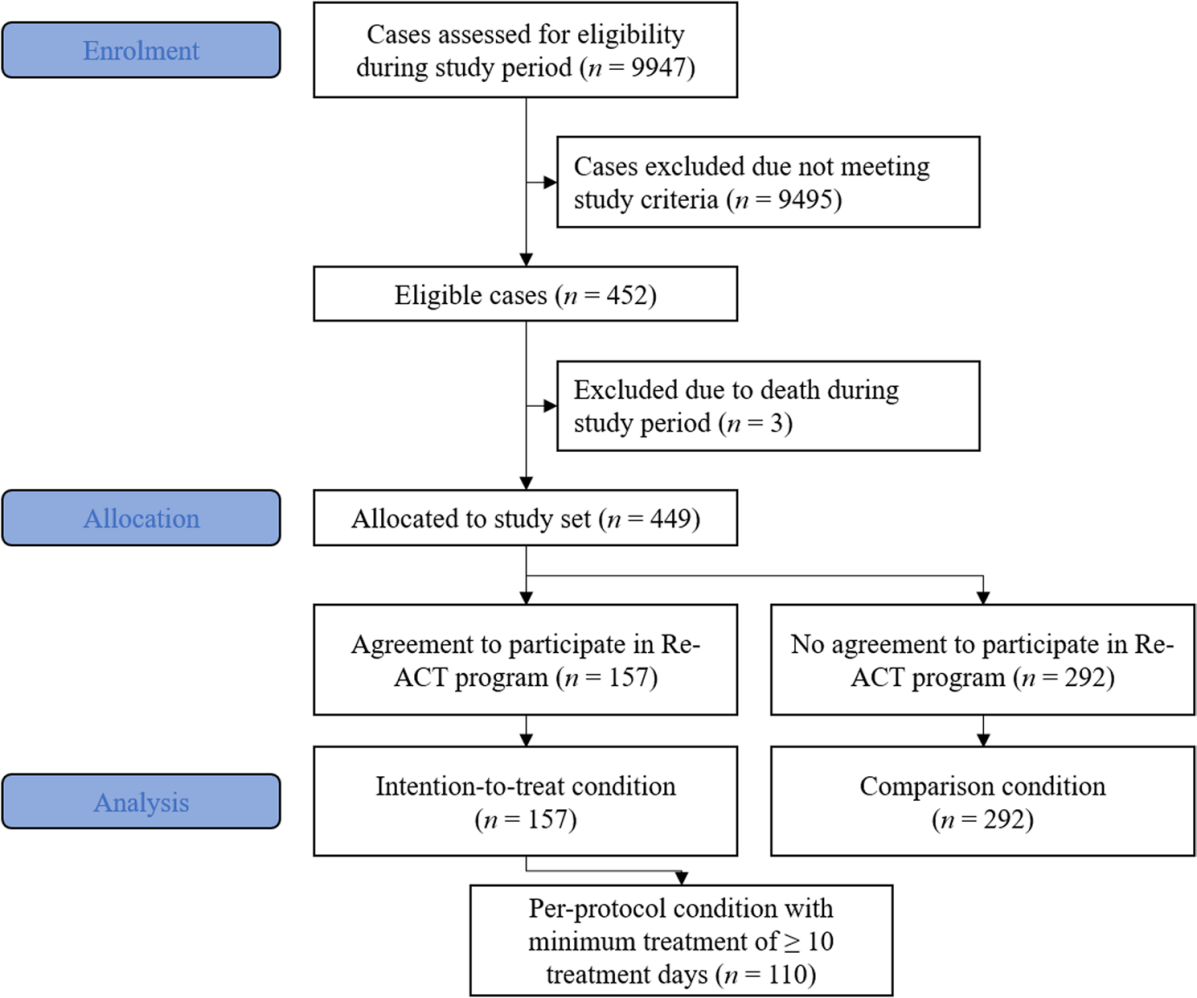
Illustration of enrolment process and data generation using an adapted CONSORT flowchart. Note: N refers to the number of cases in the study

The project team consecutively screened all inpatients during the study period and identified patients that were eligible to join the program. A message informed the respective therapist of their eligible patients and the team in the inpatient clinic was then able to discuss participation with the patient and could sign them up for Re-ACT if they wished to participate. A first meeting was then organized in the unit (cf., [6]) and all other meetings took place outside of the hospital. The first few sessions were designed to assess the patient’s situation and build a relationship with the nurse or social worker as a basis for a jointly developed and agreed upon treatment plan. If a patient identified as eligible but the project team did not receive information about acceptance of participation in the program, their case was added to the comparison group. Figure 1 illustrates the study procedure in an adapted CONSORT flowchart.

If patients were readmitted to the psychiatric hospital during the pilot study period (April 2019 – March 2022) and were still eligible, they could be invited again to join the program. Therefore, the same patient could create multiple cases in the intervention and/or comparison group in our data set.

### Design

This study’s naturalistic and non-randomized design includes the factor study condition with two groups: the *intention-to-treat* (*ITT) group* with all cases where a patient had voiced the intention to be treated and the *comparison group* with cases where no agreement to participate in the program was received by the project team. For further analyses, a *per-protocol (PP) group* was defined post-hoc containing cases in the ITT group with a minimum of 10 treatment session days (see also Fig. 1).

As dependent variables, we assessed different objective outcomes for 183 days after the admission of a patient to the ITT (PP) group or, in the case of the comparison group, after their discharge from the hospital. Our main outcome of interest was the number of inpatient treatment days in the psychiatric hospital; further objective and subjective outcome measures are described below.

### Outcome measures

#### Objective measures

Given that the individuals who were eligible to participate in the program were patients with multiple inpatient stays and therefore had an electronic file record, we were able to follow up on potential future stays recorded at the University Psychiatric Clinics Basel. To investigate the program’s effectiveness, we looked at the 183-day (6 months) period after discharge from an index stay during which they were identified as eligible to participate in the program. During the 183 days, we recorded the number of inpatient stays at the hospital, the number of days as an inpatient, and whether involuntary admissions occurred (cf., [14]). We opted for a 183-day follow-up period to ensure that treatment effects would be visible [27] and could be attributed to the program and not to other changes.

#### Subjective measures

We assessed participants’ experience in regards to the program. Previous research has indicated that patient satisfaction with the care they receive affects future treatment seeking outcomes [28]. During participation in the Re-ACT program, patients were able to provide feedback and discuss changes with the project team member in charge. After completing the program, their general satisfaction was assessed using the Münsterlingen Patient Satisfaction Questionnaire for outpatient settings ([29], in German: “Münsterlinger Fragebogen zur Patientenzufriedenheit ambulant”, short: MüPF ambulant, [30]), which is used in Switzerland (see [29, 31]). The questionnaire contains 21 items and assesses satisfaction with different components of the program. Re-ACT participants received the printed version of the questionnaire either in the last session or via the standard mail and were asked to provide anonymous feedback and send it back to the hospital.

### Data and analyses

Study data were compiled using electronic patient records, which included sociodemographic data, such as gender and age, diagnoses, and the date of the index stay and discharge or, in the case of the ITT (PP) group, the start of the Re-ACT program. We further accessed the number of previous inpatient stays at the University Psychiatric Clinics Basel overlapping with the 2.5 years (913 days, see inclusion criteria) prior to the Re-ACT starting date for cases in the ITT (PP) group or the discharge date from the index inpatient stay for cases in the comparison condition. For the follow-up period of 183 days, the number and duration of inpatient stays and involuntary admissions were added. For the ITT (PP) group, the number of sessions of assertive community treatment was recorded. In a separate data set, results regarding Re-ACT participant satisfaction were collected from the respective questionnaires returned to the project team. To integrate all data related to the Re-ACT program, we analyzed the data on the case-level. To complement these analyses, we additionally added a patient-level analysis. To do so, we first used only the first case of every patient, except for situations in which a patient who first declined program participation (and therefore created a case in the comparison group) joined the program within the 183-day observation period following the control case and therefore received treatment, potentially confounding the outcome measures. In these 8 cases, we decided to use the first case in the Re-ACT program to ensure that outcomes within the observation period can be associated with the receipt or non-receipt of the treatment. Second, we computed mixed model regression analyses [32, 33] to ensure the inclusion of all data points, and added a random intercept for participants to reflect the fact that multiple cases could be present for patients. Here, we also included further control variables to investigate the stability of the results.

All analyses reported in this manuscript were computed with R-Studio (Version 2024.12.0, [34]).

## Results

Results describe the feasibility of the program’s implementation and report preliminary findings regarding its effects during the pilot phase.

### Project team and participants

**Fig. 2:**
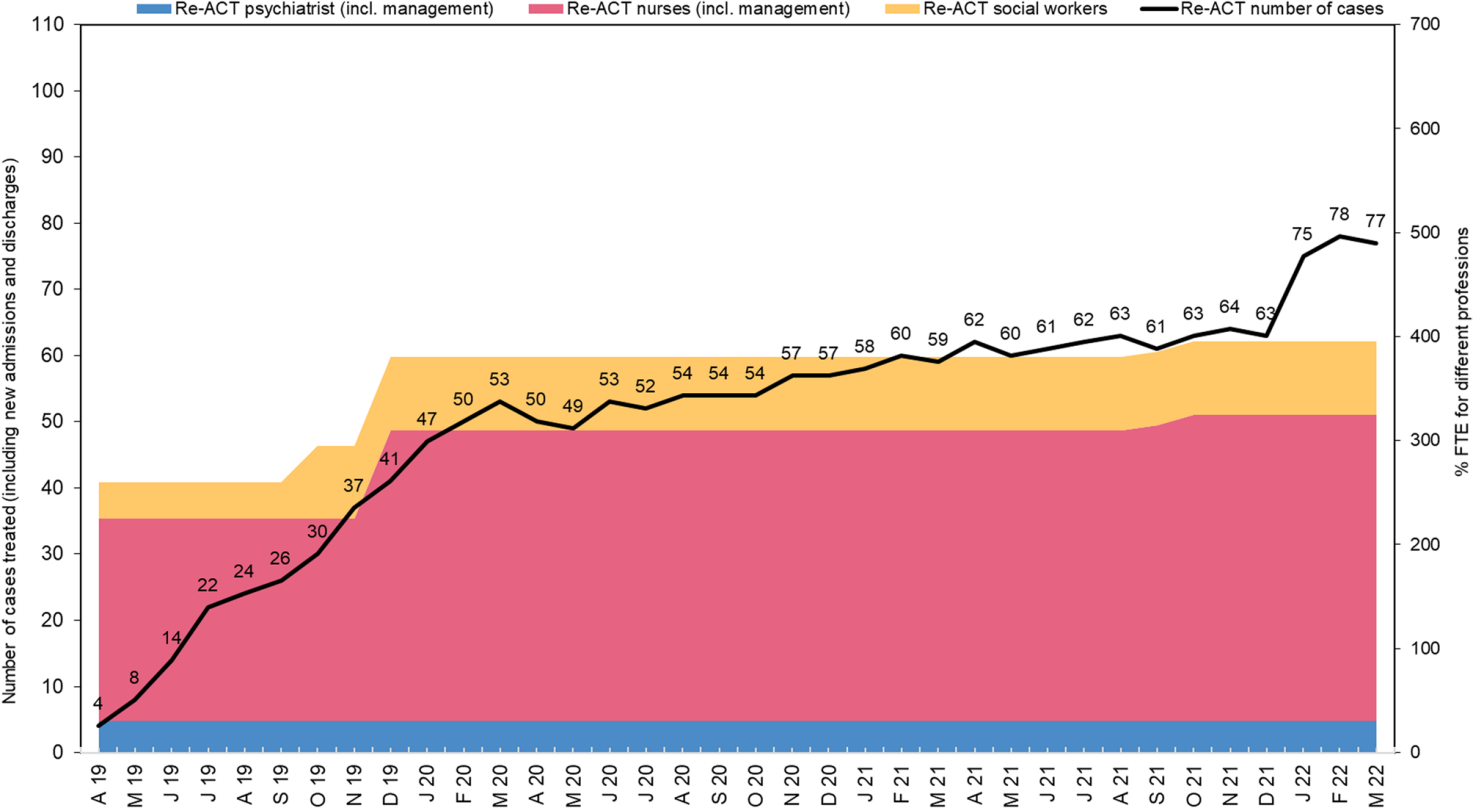
Development of the Re-ACT team and number of cases handled across the 3-year pilot project. Note. Across the 3-year pilot phase, the project started with 195% full-time equivalents (FTE) of nurses, 35% FTE of social workers, and 30% FTE of psychiatrists. At the end of the project phase, the team had expanded to 295% FTE of nurses, 70% FTE of social workers, and 30% FTE of psychiatrists. The caseload of the project started with 4 cases in April 2019 and ended with 77 active cases in March 2022

The Re-ACT program was delivered by a multidisciplinary team consisting of psychiatrists, social workers and nurses. Figure 2 illustrates the project team and the number of cases handled across the study period.

Overall, 271 patients (with 452 cases) were eligible to join the Re-ACT program (given that patients were again invited to join the project after readmission to the psychiatric hospital, one patient could create several cases within each but also between conditions). Throughout the project, 141 patients (with 160 cases) accepted the invitation and formed the ITT group. For 162 patients (with 292 cases) no acceptance was received and they formed the comparison group.

Importantly, given the eligibility criteria for the program, many of the participants in this study suffered from severe mental illnesses. Over the course of the study program, 3 participants died (including one suicide) in the ITT group (we lack precise data for the comparison group as contact was limited). For the analyses below, we excluded their data.

We further investigated the ITT group and analyzed the amount of assertive community treatment that participants had received using the days with billable treatment units as a proxy. On average, participants had 25.99 (*SD* = 19.25) days with treatment, however, this number ranged from 1 to 74 with 5 participants with no information and presumably zero appointments. Arguing that a minimum level of treatment is required for the program to be effective, we used a cut-off value of a minimum of 10 days with treatment appointments to create a *PP group* (cf. [35], for a similar approach). This PP group consisted of 104 patients with 110 cases; see also Fig. 1. For further details on the cases in the ITT, PP, and comparison group, see Table 1.

**Table 1:** Descriptive overview of cases’ gender, age, and diagnosis count in different conditions

Variable		Overall	ITT group	PP group	Comparison group
Gender	Male	197 (43.88%)	58 (36.94%)	46 (41.82%)	139 (47.60%)
	Female	236 (52.56%)	90 (57.32%)	61 (55.45%)	146 (50.00%)
	no information	16 (3.56%)	9 (5.73%)	3 (2.73%)	7 (2.40%)
Age (years)	*M (SD)*	42.58 (11.31)	44.74 (11.94)	45.26 (12.44)	41.46 (10.82)
Diagnosis (ICD-10)	F0X/Organic, including symptomatic, mental disorders	4 (0.89%)	2 (1.27%)	1 (0.91%)	2 (0.68%)
	F1X/Mental and behavioral disorders due to psychoactive substance use	60 (13.36%)	8 (5.10%)	5 (4.55%)	52 (17.81%)
	F2X/Schizophrenia, schizotypal and delusional disorders	141 (31.40%)	43 (27.39%)	36 (32.73%)	98 (33.56%)
	F3X/Mood/affective disorders	108 (24.05%)	41 (26.11%)	33 (30.00%)	67 (22.95%)
	F4X/Neurotic, stress-related and somatoform disorders	31 (6.90%)	13 (8.28%)	12 (10.91%)	18 (6.16%)
	F5X/Behavioral syndromes associated with physiological disturbances and physical factors	1 (0.22%)	0 (0%)	0 (0%)	1 (0.34%)
	F6X/Disorders of adult personality and behavior	68 (15.14%)	26 (16.56%)	18 (16.36%)	42 (14.38%)
	F8X/Disorders of psychological development	4 (0.89%)	1 (0.64%)	1 (0.91%)	3 (1.03%)
	Other/No information	32 (7.13%)	23 (14.65%)	4 (3.64%)	9 (3.08%)
Number of previous inpatient stays	*M (SD)*	7.09 (5.58)	6.17 (4.23)	5.79 (3.82)	7.59 (6.13)
	*Md*	5.00	5.00	5.00	6.00

Age was computed as the difference in years between cases’ date of birth and the start date of the recovery-oriented assertive community treatment program for cases in the ITT (intention-to-treat) and PP (per-protocol) condition and the discharge date of the index stay for cases in the comparison condition. For 16 cases, missing values were recorded. The diagnoses represent the main diagnosis coded at the time the patient was included in the intervention or comparison group. The number of previous inpatient stays was computed by counting all overlapping inpatient stays at the University Psychiatric Clinics Basel that occurred during the 2.5 years (913 days) prior to the Re-ACT starting date for cases in the ITT (PP) group or the discharge date from the index inpatient stay for cases in the comparison condition

### Objective measures

Table 2 summarizes the objective results. Looking at differences in average outcomes, we find 42.34% fewer inpatient days, 31.85% fewer inpatient stays and 78.26% fewer involuntary admissions when comparing the PP and comparison cases. For the ITT versus the comparison cases, we find 29.38% fewer inpatient days, 25.48% fewer inpatient stays and 78.26% fewer involuntary admissions.

**Table 2:** Descriptive results of outcome variables overall and separately for cases in the different conditions

	Statistic	Target variable observed during follow up period of 183 days		
		Number of inpatient treatment days in psychiatric hospital	Number of inpatient stays at the psychiatric hospital	Number of involuntary admissions to the psychiatric hospital
Overall (*n* = 449)	*Min - Max*	0–179	0–14	0–9
	*M (SD)*	24.71 (37.05)	1.43 (1.87)	0.16 (0.63)
	*25% P*	0.00	0.00	0.00
	*Md*	7.00	1.00	0.00
	*75% P*	33.00	2.00	0.00
PP group (*n* = 110)	*Min - Max*	0–129	0–8	0–2
	*M (SD)*	15.88 (26.47)	1.07 (1.70)	0.05 (0.25)
	*25% P*	0.00	0.00	0.00
	*Md*	0.00	0.00	0.00
	*75% P*	22.75	1.75	0.00
ITT group (*n* = 157)	*Min - Max*	0–152	0–8	0–2
	*M (SD)*	19.45 (32.38)	1.17 (1.75)	0.05 (0.25)
	*25% P*	0.00	0.00	0.00
	*Md*	0.00	0.00	0.00
	*75% P*	26.00	2.00	0.00
Comparison group (*n* = 292)	*Min - Max*	0–179	0–14	0–9
	*M (SD)*	27.54 (39.09)	1.57 (1.92)	0.23 (0.76)
	*25% P*	0.00	0.00	0.00
	*Md*	10.00	1.00	0.00
	*75% P*	38.00	2.00	0.00

ITT is the abbreviation for the intention-to-treat condition and PP the abbreviation for the per-protocol condition

To compare outcomes between cases in the ITT, PP, and comparison group, we computed Wilcoxon tests. All comparisons between the PP and the comparison group were significant; *W* = 12,641, *p* <.001, *r* =.17 for inpatient treatment days, *W* = 12,696, *p* <.001, *r* =.17 for inpatient stays, and *W* = 14,275, *p* =.002, *r* =.15 for involuntary admissions. Comparisons remained significant when using the ITT group; *W* = 19,027, *p* =.002, *r* =.15 for inpatient treatment days, *W* = 18,930, *p* =.001, *r* =.15 for inpatient stays, and *W* = 20,547, *p* <.001, *r* =.16 for involuntary admissions.

We complemented this case-level analysis by looking at individual-level data, using only one case per patient. This procedure, however, resulted in a loss of data and reduced the data set to 268 patients/cases, with 119 patients in the ITT group and 149 patients in the comparison group. We then recomputed the objective outcome measures. The ITT group had an average of *M* = 17.61 inpatient days (*SD* = 31.10, *Md* = 0), *M* = 1.02 inpatient stays (*SD* = 1.72, *Md* = 0), and *M* = 0.05 involuntary admissions (*SD* = 0.26, *Md* = 0). The comparison group had an average of *M* = 20.60 inpatient days (*SD* = 33.04, *Md* = 4), *M* = 1.08 inpatient stays (*SD* = 1.45, *Md* = 1), and *M* = 0.16 involuntary admissions (*SD* = 0.49, *Md* = 0). Group comparisons using Wilcoxon tests resulted in non-significant findings for differences in inpatient days (*W* = 7907, *p* =.104, *r* =.10), inpatient stays (*W* = 7910, *p* =.102, *r* =.10), but significant findings for the number of involuntary admissions (*W* = 8219.5, *p* =.031, *r* =.13).

We recomputed this analysis for the data from the PP group. Here, changing to an individual focused analysis resulted in reducing the data to 235 patients/cases, with 86 patients in the PP group and 149 patients in the comparison group. The PP group had an average of *M* = 14.09 inpatient days (*SD* = 25.46, *Md* = 0), *M* = 0.95 inpatient stays (*SD* = 1.67, *Md* = 0), and *M* = 0.05 involuntary admissions (*SD* = 0.26, *Md* = 0). Group comparisons using Wilcoxon tests resulted in non-significant findings for differences in inpatient stays (*W* = 5576, *p* =.074, *r* =.12), but significant findings for differences in inpatient days (*W* = 5484, *p* =.0497, *r* =.13) and number of involuntary admissions (*W* = 5898.5, *p* =.036, *r* =.14).

In order to control for the repeated assessment of individuals in our data without losing a large number of cases (see above), we also used regression models for each outcome variable to analyze differences between conditions. Given the count-based nature of our outcomes, we log-transformed all outcome variables and computed a mixed regression model with participant ID as a random effect and condition as a fixed effect (PP vs. comparison group, with the PP as reference category). Results show that condition remained a significant predictor for the number of inpatient days (*b* = 0.55, *SE b* = 0.19, *t*(393.72) = 2.82, *p* =.005) and involuntary admissions (*b* = 0.09, *SE b* = 0.03, *t*(393.60) = 2.94, *p* =.004), but did not reach significance when investigating the number of inpatient stays (*b* = 0.12, *SE b* = 0.07, *t*(397.32) = 1.72, *p* =.087). This pattern is stable when looking at the ITT group, too, with *b* = 0.39, *SE b* = 0.17, *t*(445.63) = 2.26, *p* =.024 for inpatient days, *b* = 0.09, *SE b* = 0.03, *t*(439.35) = 3.12, *p* =.002 for involuntary admissions, and *b* = 0.08, *SE b* = 0.06, *t*(446.75) = 1.33, *p* =.184 for inpatient stays.

We further tested whether conditions differed in regards to the number of previous inpatient admissions (see Table 1). This was the case when comparing the PP and the comparison condition, *W* = 11,843, *p* <.001, *r* =.15, and the ITT and the comparison condition, *W* = 18,166, *p* <.001, *r* =.17. Given this significant difference, we included the number of previous inpatient admissions as a control variable and fixed effect into the above-described regression models. Results indicate that condition remains significant for the number of inpatient treatment days (*b* = 0.49, *SE b* = 0.19, *t*(393.63) = 2.56, *p* =.011), and number of involuntary admissions (*b* = 0.09, *SE b* = 0.03, *t*(394.1) = 2.85, *p* =.005), but condition again did not reach significance when looking at the number of inpatient stays (*b* = 0.10, *SE b* = 0.07, *t*(349.9) = 1.51, *p* =.132). As described in the analyses above, the pattern again remains stable when looking at the ITT group.

### Subjective measures

Fifteen participants returned the questionnaire assessing satisfaction. Overall, participants who provided feedback appeared very satisfied with the program (Item 17), *M* = 6.20, *SD* = 1.21. Computing a satisfaction score across all 21 items resulted in an average score of 5.94, *SD* = 0.88. For a rough comparison, Billian and colleagues [31] reported an overall mean score of 4.6 using a similar questionnaire among patients who received outpatient treatment from the same hospital.

Table 3 summarizes the descriptive results from the patient satisfaction questionnaire on the item level. Item 7 was recoded as the question was framed negatively, whereas all other items were framed positively. Patients were most satisfied (*M* > 6.50) with the respectful treatment by the staff and described feeling well cared for when they had physical problems (see Table 3). Patients were least satisfied with the clarity of explanations of the medication and potential side effects (Item 10), *M* = 4.60, *SD* = 1.43.

**Table 3:** Patient satisfaction with the recovery-oriented assertive community treatment program at program completion, assessed with the Münsterlingen patient satisfaction questionnaire

	Item	*n*	Min	Max	Mean	SD
1	The service is easy to reach by telephone.	12	3	7	6.17	1.27
2	I was able to adequately explain my situation in the initial consultation.	14	3	7	6.07	1.38
3	After joining the program, the next steps were explained to me.	15	4	7	6.20	1.15
4	I felt treated with respect by the clinical staff.	15	4	7	6.67	0.90
5	When I am in distress, I know where to turn.	15	4	7	6.40	0.83
6	I trust the people who are treating me.	15	3	7	6.40	1.12
7	I was hesitant to ask questions. (R)	15	1	7	4.87	2.20
8	Treatment goals were agreed with me.	12	4	7	5.42	1.16
9	I was able to influence the planning of my treatment.	15	3	7	5.87	1.25
10	The effects of the medication and possible side effects were clearly explained to me.	10	3	7	4.60	1.43
11	I was able to influence the medication treatment.	10	3	7	5.40	1.71
12	The service providers had enough time to talk to me.	15	4	7	6.00	1.25
13	I was supported and accompanied in my search for other sources of support (including agencies, self-help groups).	12	4	7	6.33	0.98
14	The collaboration between my relatives and those treating me met my needs.	7	4	7	6.00	1.15
15	How helpful did you find working with your caregiver?^a^	14	4	7	6.00	1.11
16	The treatment helped me to better deal with my problems.	15	2	7	5.40	1.84
17	Overall, I am satisfied with my treatment.	15	3	7	6.20	1.21
18	I would recommend this treatment offer.	15	3	7	6.33	1.29
19	I feel well cared for when I have physical problems.	11	4	7	6.55	0.93
20	My follow-up care after the assertive community treatment was sufficiently organized.	14	3	7	6.43	1.28
21	Since starting treatment with the assertive community treatment program, my condition is now^b^	15	1	7	4.87	1.64

Replies were recorded on a Likert-scale ranging from 1 = *does not apply at all* to 7 = *fully applies*, with an additional option of 8 = *cannot answer* (coded as a missing for the data analysis)

^a^ The labels of the Likert scale for this item were 1 = *not helpful at all* to 7 = *very helpful*

^b^ The labels of the Likert scale here were 1 = *much worse* to 7 = *much better*

Participants further provided open comments and mentioned “Talking at eye level, the provider’s competence and empathy”, the possibility to receive treatment “at home in well-known surroundings” and “being supported to gain ground in one’s everyday life” positively.

## Discussion

The results from the program’s three-year pilot phase provides support for the feasible and successful implementation of recovery-oriented assertive community treatment in Basel-Stadt. This Re-ACT program offers patients with a history of high utilization of inpatient treatment a need-adapted treatment option that may support them during their discharge and recovery at home for as long as required. The multidisciplinary team started with 4 cases at the beginning of the project and had more than 70 active cases at the end of the pilot project.

Looking at objective and subjective outcome measures, the results from the feasibility study provide a potentially promising outlook: The number of days as an inpatient, the number of inpatient stays, and the number of involuntary admissions during a 183-day follow-up period were lower when comparing cases in the treatment versus comparison group. This was true for the cases in the ITT group (which included all cases with participants who agreed to participate in Re-ACT) as well as cases in the PP group (which required a minimum number of 10 treatment days). Including further control variables showed that this finding is stable regarding inpatient days and involuntary admissions, but that differences regarding inpatient stays do not reach significance. Complementing additional patient-level analyses with only one case per patient, however, indicated that only the differences in the number of involuntary admissions remained significant across analyses. Looking at the subjective outcome measures, the small sample of participants who completed the program and returned feedback questionnaires reported high levels of satisfaction.

These results are in line with previous research showing that assertive community treatment can be successfully introduced and may positively affect both objective [14, 36, 37] and subjective [35] outcomes of people with severe mental illnesses (for a general review, see [9]). As individual recovery goals can be taken into account and patients are supported in their community and home [38], the Re-ACT program may have the potential to successfully fill a treatment gap in the realm of mental health.

### Limitations

Although the presented results support the introduction of the program and suggest positive preliminary findings that await further replication, limitations apply. Our objective outcome measures regarding inpatient stays only focus on stays at the University Psychiatric Clinics Basel. While the hospital is the main healthcare provider for persons with a severe mental illness in the region and most of the treatments presumably take place there, there might have been additional stays in other hospitals that were not recorded for this analysis. The subjective measure, furthermore, relies on self-reports and suffers from a low response rate (presumably due to the way the questionnaires were collected) and a self-selected and potentially biased subsample of program participants who returned the feedback questionnaire. The outcomes may therefore not generalize to all program participants.

Furthermore, the study builds on a non-randomized design where participants with their inpatient health care team could choose whether they wanted to participate in the program. Whereas this naturalistic design may speak to the ecological validity of the effects of such a program, it reduces the internal validity of the study as self-selection biases are likely present. Cases in the intention-to-treat and the per-protocol condition may differ from cases in the comparison condition (see, e.g., the significant differences in the number of previous inpatient stays), and these differences may affect the outcome variables. Furthermore, the same patient, if readmitted during the study period, could create several cases within and across conditions. This is problematic insofar as it results in a violation of the assumption of independence of observations in the data (see, e.g., [39]). When applying different analytic strategies to reinvestigate differences in outcomes while controlling for repeated measurement of the same patient (by using mixed models or only including one case per patient), the result replicated only in part. This (for the latter analysis) might be a result of the differences in sample size and statistical power or may indicate that the preliminary results are not fully robust. Further assessment of the program’s effect with a larger set of data is therefore required and independent replication recommended.

Replication studies could also benefit from design improvements. Future studies should aim for a randomized control setup [16, 40], include additional control variables or use a case-by-case matching procedure. Further, a-priori rules for data collection and data use from patients who are readmitted during the study period and/or have not received a minimum amount of treatment, may be specified. Future research may also look at longer observation periods (see, e.g., [36]) as well as additional outcome measures (e.g., self-reported individual needs or quality of life, [41]) and use different analysis methods (e.g., survival analysis) to increase our understanding of if, when, and how outreach programs may benefit participants.

Finally, our Re-ACT program was designed to provide support after an inpatient stay for people with severe mental illnesses and a history of hospitalizations. It comprises a specific setup of multidisciplinary teams and a certain level of support. As described above, assertive community treatment programs can be designed very differently and may include many different components. Our results may therefore speak for the feasibility of our setup and further research is warranted to test whether different setups may result in different outcomes.

### Outlook and conclusion

After March 2022, the pilot Re-ACT project was extended for another three years. Initial adjustments have been made in waiving the upper age limit and addiction related diagnosis restrictions for participation. Summarizing the first phase, we conclude that the Re-ACT program was implemented successfully and provides need-adapted and recovery-oriented treatment for people with a severe mental illness. Program participation was descriptively associated with fewer days in inpatient care, fewer inpatient stays, and fewer involuntary admissions in the follow-up period. These differences were significant using a case-level analysis strategy. An individual-level strategy (associated with loss of data) only replicated significant differences for involuntary admissions. Furthermore, selection effects seem to be present due to the naturalistic design of the study. Feedback regarding satisfaction with the program was very positive. Although the different effects regarding the objective outcome measures await further replication, the program is feasible and seems to offer the opportunity to provide an important building block in individualized integrated care in Switzerland.

## Data Availability

The data is not publicly available but an anonymized version of the here presented variables can be shared upon request to any qualified researcher.
